# In Memoriam—Dr. Robert Battey

**Published:** 1896-03

**Authors:** James B. Baird, Virgil O. Hardon, J. B. S. Holmes

**Affiliations:** Committee. Atlanta Obstetrical Society. Atlanta, Ga.; Committee. Atlanta Obstetrical Society. Atlanta, Ga.; Committee. Atlanta Obstetrical Society. Atlanta, Ga.


### IN MEMORIAM—DR. ROBERT BATTEY.

ATLANTA OBSTETRICAL SOCIETY.
ATLANTA, GA., January 15, 1896.

In the recent death of Dr. Robert Battey, of Rome, Ga., the State, the country, and the profession at large have lost one of their most striking and remarkable men. Probably no surgeon of modern times, with the exception of Lister, has left a more radical and enduring impress upon surgical practice. His advent into the field of abdominal surgery occurred at a time when this branch of practice was confined to the then doubtful operation of ovariotomy for ovarian cysts, and when a mortality of twenty-five per cent. was considered an excellent record. At his death the hand of surgery had invaded every organ and every tissue in the abdomen, and the mortality, in the hands of skilled operators, had been reduced below five per cent. This remarkable change can be largely traced to the inception and introduction of Battey's operation. It is doubtless true that the time was ripe for this extension of abdominal surgery. It was in the air, so to speak, and if Battey had not caught the idea some other man would, but this fact in no wise detracts from the originality and boldness of this innovation in surgery. The debt which the profession owes him is therefore to be measured by the advance which abdominal surgery has made in the last score of years.

Dr. Battey's claim to fame is founded upon the operation which bears his name. But, even aside from this, he possessed many of the elements of greatness. He was an indefatigable worker, an eloquent speaker, and a man who had the courage of his convictions. He was withal a modest man, and never sought personal aggrandizement. He forced his operation upon the attention of the profession for its own sake, and not from any selfish motive. With all the honors which came to him in his later years, he was content to live in comparative obscurity in the little town which had been the scene of his earlier struggles, intent only on the good which he might do to his fellow-men. In all the relations of life he was an ideal man, and he lived upon a high plane, which separated him from his fellows without taking him outside of the sphere of their sympathies and companionship. In his death we feel that a great man has departed from among us, and the void which is left is one which will not easily be filled. But his memory will live as long as the profession of medicine remains, and his name will be mentioned along with the greatest surgeons which the world has seen. As long as there are suffering women who seek relief, so long will the name of Battey be held in lasting honor and reverence.

JAMES B. BAIRD, M.D.,
VIRGIL O. HARDON, M.D.,
J. B. S. HOLMES, M.D.,
Committee.

